# Supplementary material for: A Triazole Hybrid of Neolignans as a Potential Antileishmanial Agent by Triggering Mitochondrial Dysfunction
Source: Molecules. 2019 Dec 20;25(1):37. doi: 10.3390/molecules25010037 (PMC6983227; doi:10.3390/molecules25010037)
Supplement: Supplementary file 1 [file molecules-25-00037-s001.pdf]

Supporting information

A triazole hybrid of neolignans as a potential antileishmanial agent by triggering mitochondrial dysfunction

Carla Cardozo Pinto de Arruda[1,*], Daiana de Jesus Hardoim[2], Yasmin Silva Rizk[2], Celeste da Silva Freitas de Souza[2], Tânia Zaverucha do Valle[2], Diego Bento Carvalho[3], Noemi Nosomi Taniwaki[4], Adriano Cesar de Morais Baroni[3] and Kátia da Silva Calabrese[2,*]

[1] Laboratório de Parasitologia Humana, Instituto de Biociências, Universidade Federal de Mato Grosso do Sul, 79090-900 Campo Grande-MS, Brasil.

[2] Laboratório de Imunomodulação e Protozoologia – LIMP, Instituto Oswaldo Cruz - IOC, Fundação Oswaldo Cruz – FIOCRUZ, 21040-900 Rio de Janeiro-RJ, Brasil.

[3] Laboratório de Síntese e Química Medicinal - LASQUIM, Faculdade de Ciências Farmacêuticas, Alimentos e Nutrição, Universidade Federal de Mato Grosso do Sul, 79090-900 Campo Grande-MS, Brasil.

[4] Núcleo de Microscopia Eletrônica, Instituto Adolfo Lutz, 01246-000 São Paulo-SP, Brasil.

* Authors to whom correspondence should be addressed: carla.arruda@ufms.br; Tel.: +55-67-3345-7369; kscalabrese@gmail.com; Tel.: +55-21-2562-1879.

Received: date; Accepted: date; Published: date.

List of contents

General Remarks 1

1. Preparation of triazole **6** (LASQUIM 25) 2
2. Spectra data triazole **6** – ^{1}H NMR, ^{13}C NMR, HRMS....................................2
3. References..2

General Remarks

Anhydrous solvents were dried and distilled before use according to the standard procedure. All chemicals for synthesis were of reagent grade and used without purification unless noted. Reactions were carried out under a nitrogen atmosphere and monitored by TLC using prepared plates (silica gel 60 F254 on aluminum). The chromatograms were examined under both 254 and 360 nm UV light or with the developing agent ethanolic vanillin and heat. Flash column chromatography was performed on silica gel 60 (particle size 200-400 mesh ASTM, purchased from Aldrich, USA). Melting points were determined using Fisatom 430D equipment. The ^{1}H and ^{13}C NMR spectra were recorded in $CDCl_3$ solutions using a Brucker 75 MHz or 300 MHz spectrometer, as noted. Chemical shifts (δ) are expressed as parts per million (ppm) downfield from tetramethylsilane or deuterated solvent ($CDCl_3$ ^{1}H δ 7.27 and ^{13}C δ 77.0 ppm; DMSO-D_6 ^{1}H δ 2.5 and ^{13}C δ 39.51 ppm; Acetone ^{1}H δ 2.05 and ^{13}C δ 77.0 ppm) as the internal standard. HR-ESI-MS measurements were carried out on a quadrupole time-of-flight instrument (UltrOTOF-Q, BrukerDaltonics, Billerica, MA).

*1. Preparation of triazole **6** LASQUIM 25*

Figure 1. Synthesis of the triazole **6** (LASQUIM 25).

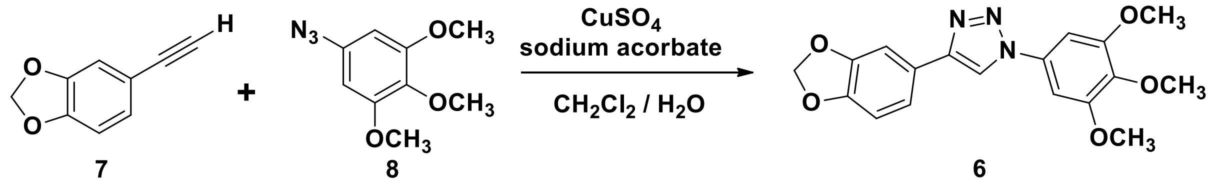

General Method [2]:

It was added $CuSO_4.5H_2O$ pentahydrate (1.0 mmol, 0.249g) and sodium ascorbate (2.0 mmol, 0.396g) to a solution of terminal acetylene **7** (10 mmol, 1.46g) and azide **8** (10 mmol, 2.09g) in dichloromethane (10 mL) and water (10 mL). The reaction mixture was stirred for 24 h. Then was added a saturated solution of NH_4Cl (150 mL) and the product was extracted with dichloromethane (3 x 50 mL). The organic phase was dried over anhydrous $MgSO_4$, and the solvent was evaporated under reduced pressure. The product was purified by recrystallization from ethyl acetate affording **6** (LASQUIM 25) in 80% yield (1.42g).

*2. 4-(benzo[d][1,3]dioxol-5-yl)-1-(3,4,5-trimethoxyphenyl)-1H-1,2,3-triazole **6***

Spectra data in accordance with ref. [2]: ^{1}H NMR (300 MHz, $CDCl_3$) δ 3.87 (s, 3H, OCH_3), 3.92 (s, 6H, $2OCH_3$), 5.99 (s, 2H, CH_2), 6.86 (d, 1H, *J* 8.0 Hz, Ar-H), 6.96 (s, 2H, Ar-H), 7.36 (m, 2H, Ar-H), 8.02 (s, 1H, *Tr-H); ^{13}C NMR (75 MHz, $CDCl_3$) δ 56.43, 61.03, 98.45, 101.24, 106.43, 108.70, 117.27, 119.57, 124.32, 132.89, 138.32, 147.81, 148.18, 153.91. HRMS (ESI+) *m/z* calcd. for $C_{18}H_{17}N_3O_5$ [M + H]+: 356.1246; found: 356.1253; *Tr: triazole hydrogen.

3. References

[1] Amarego, W. L. F.; Perrin, D. D. *Purification of laboratory chemicals*, 3th ed., Pergamon Press: New York, 1988.

[2] Cassamale, T.B.; Costa, E.C.; Carvalho, D.B.; Cassemiro, N.S.; Tomazela, C.C.; Marques, M.C.S.; Ojeda, M.; Matos, M.F.C.; Albuquerque, S.; Arruda, C.C.P.; Baroni, A.C.M. Synthesis and antitrypanosomatid activity of 1,4-Diaryl-1,2,3-triazole analogues of neolignans veraguensin, grandisin and machilin G. *J. Braz. Chem. Soc.* **2016**, *27*, 1217-1228.
